# Optimization of fermentation media and growth conditions for microbial xylanase production

**DOI:** 10.1007/s13205-016-0445-3

**Published:** 2016-06-03

**Authors:** Bushra Kalim, Nazish Mazhar Ali

**Affiliations:** Microbiology Laboratory, Department of Zoology, GCU, Lahore, Pakistan

**Keywords:** Xylanase, Characterization, Xylanolytic microbes, Enzymatic activity

## Abstract

Efficiency of cellulase-free xylanases is one of the determining factors in paper and pulp industries. Use of microbes which can produce cellulase-free xylanases may help to overcome the current challenges in kraft pulp processing. Isolation and screening of microorganisms from local samples offers a possibility for obtaining the potential microbes for this purpose. This research was therefore aimed to collect, screen, characterize and identify potential cellulase-free xylanase producers. A total of 313 microbial isolates were collected while using selective media (EBAM and XAM) to determine the xylanolytic potential of microbes. Qualitative and quantitative analyses were performed and finally 11 bacterial and 6 fungal strains were selected for characterization and identification. The potential isolates were identified *as Bacillus pumilus* (388.82 U/mg), *Bacillus safensis* (385.26 U/mg), *Aspergillus flavus* (493.33 U/mg) and *Aspergillus niger* (419.33 U/mg). Optimization of the microbial strains while using agro-industrial waste is suggested.

## Introduction

Xylanases are of great importance in a number of industrial processes and the increasing trend towards an environment friendly industrialization has paved its ways. Hemicelluloses, being the second most abundant component of plant biomass tend to be suitable agro-industrial residue (Collins et al. 2005). 20–35 % of the total dry mass of plants constitutes xylans which are useful as fermentation substrate for the production of sugars and biofuel (Haltrich et al. 1996; Filho 1998). Xylans are heterogeneous compounds and require synergistic reaction of hydrolytic enzymes for their complete degradation. Among these enzymes, endo-β-1,4-xylanases (EC.3.2.1.8) are the most important xylanolytic enzymes which cleave off the internal glycosidic bonds in xylan backbone, reducing the degree of polymerization (Biely 1985; Polizeli et al. 2005). The cleavage carried out by these enzymes is very specific and the degree of substitution in the polymer affects the hydrolysis product (Dodd and Cann 2009).Interest in the xylanolytic enzymes has received much attention due to their potential uses in different industrial processes such as kraft pulp bleaching, biopulping in paper and pulp industry, in animal feed stock, bread and beverages (Kirk and Yang 1979; Schwien and Schmidt 1982; Viikari et al. 1986; Madlala et al. 2001).

Some marine algae are also reported to secrete xylanases in extracellular environment (Polizeli et al. 2005). Xylanases of protozoan and crustacean origin are also reported (Devillard et al. 1999; Polizeli et al. 2005). Unusual xylanases from the gut of insects have been studied (Roy et al. 2003; Brennan et al. 2004). Wu and He (2015) have recently reported isolation of a xylanolytic strain *Cellvibrio mixtus* from giant snail in Singapore whereas similar reports of xylanases from fresh water mollusc have been noted from Yamura (Yamura et al. 1997). Many microbial sources including bacteria, fungi and yeast are being reported with tolerable temperature and pH ranges (Sunna and Antranikian 1997). Filamentous fungi, especially from *Aspergillus* and *Trichoderma* spp. are reported to be the best sources of xylanases with higher levels of extracellular xylanase production (Haltrich et al. 1996).

To contribute in the improvement of biodegradation of hemicelluloses through microbial co-cultures, exploration, selection and characterization of potential cellulase-free xylanase producing microbes may have important roles. The production of cellulase-free xylanase is of crucial importance in paper and pulp industry. Thus, the objective of the present study was to collect, screen, identify and characterize cellulase-free xylanase producing locally isolated microbial strains.

## Materials and methods

### Sample collection and microbial isolation

Samples for isolation of xylanase producing bacteria and fungi were obtained from particular sites. Samples of soil, leaf compost, grazer dunks and paper industry waste were put into plastic bags. Fresh ruminal fluids were collected from local slaughter houses and after filtration through a muslin cloth stored at 4 °C until further use.

To isolate bacteria and fungi from soil and litter, 1 g of each crushed-sample and 1 ml of ruminal sample were suspended in 100 ml of sterile water by vortexing. Using standard spread plate technique, 100 µl of each sample suspension was spread onto the surface of nutrient agar plates supplemented with 15 ppm cycloheximide to isolate bacterial strains. For the isolation of fungal strains, 1 ml of each suspension was spread on YpSs agar plates supplemented with 0.08 % streptomycin. All plates were incubated overnight at 37 °C (for bacteria) and at 28 °C for 3–4 days (for fungi). Based on their appearance and morphology, various colonies were selected to obtain pure cultures on NA and PDA plates for bacterial and fungal isolates, respectively.

### Screening of xylanase producing bacterial

Two step screening assay was used to determine the xylanase producing potential of the microbial strains. For the initial screening of lignocelluloses hydrolytic enzyme production, the isolates were grown on wheat bran agar medium (Composition: g/L; wheat bran powder_50.0, peptone_10.0, phosphate buffer_2 ml, agar_20.0) plates to limit the number of isolates for screening assay. All purified strains were spot tested by incubating them for 2–3 days at 28 °C on WBA plates. Strains showing significant growth on WBA were selected for xylanase screening assays. To detect the xylanase production ability, microbial strains were inoculated on 0.1 % xylan agar medium (Composition: g/L; yeast extract_3.0, peptone_1.5, NaCl_3.5, NaNO_3__1.0, KH_2_PO_4__1.0, MgSO_4_·7H_2_O_0.3 Agar_20 and 0.1 % beechwood xylan) plates (pH 5.5). Plates were incubated at 28 °C ± 2 for 72 h. All the plates were stained with 0.5 % Congo red dye for about half an hour and were then destained using 1 M NaCl solution at room temperature. Zones of clearance were observed for presence of xylanase activity. Microbes showing positive results were selected for further analysis.

### Xylanase production in submerged fermentation

Xylanase activities of the selected isolates were measured using 3, 5-dinitrosalicylic acid (DNS) method (Ghose 1987) for determination of the amount of reducing sugars released during certain times of reaction mixture. Prior to xylanase assay, both the bacterial and fungal isolates were cultured in xylan broth medium (XBM) under submerged fermentation conditions (Samantha et al. 2011). For that purpose, 50 ml XBM (Composition: g/L; yeast extract_5.0, peptone_1.0, NaNO_3__1.0, KH_2_PO_4__1.0, MgSO_4_·7H_2_O_0.02, xylan_10.0) (pH 5.5) was prepared in 250 mL flask for each microbial strain separately. Medium was autoclaved at 121 °C, 15 lb for 15 min. Each strain was inoculated in separate flask and, flasks were incubated in a shaking incubator at 31 ± 2 °C for 7 days.

After 7 days of incubation, crude enzyme was extracted by filtration and the filtrate was centrifuged at 10,000 rpm for 20 min at 4 °C. The cell free culture filtrate (CFCF) was used as crude enzyme. Afterwards, the enzyme extract was filtered through 0.45 µm microfilter membranes to make it free of cellular mass. Pellets were oven dried at 70 °C overnight and weighed to record biomass.

### Well plate diffusion assay

Well plate diffusion assay was performed to qualitatively analyze enzyme activity. Xylan agar medium (Composition: g/L; xylan_9.0, agar_20.0, 0.1 M phosphate buffer_to raise volume upto1000 ml) prepared in phosphate buffer (pH 5.5) was autoclaved for 15 min at 121 °C and 15 lb (Samantha et al. 2011). Medium was poured in each disposable petri plate and was allowed to solidify. Upon solidification, wells were made by using sterilized cork borer of 10 mm diameter. To each well, 100 µl of the CFCF was poured and incubated at 31 °C overnight. To compare the effect of endo-xylanase by simple diffusion and Congo red assay, zones of clearance produced in incubated plates were recorded before and after being stained with 0.5 % Congo red dye for 30 min. Afterwards, stained plates were flooded with 1 M NaCl for destaining. Here, appearance of clear zones confirmed the presence of 1-4-β-endo-xylanase activity.

### Xylanase assay

Xylanase assay was measured according to Bailey et al. (1992). CFCF was used as crude enzyme and 300 µl was incubated with 700 µl of 1 % solubilized birchwood xylan solution at 50 °C for 30 min in shaking incubator. Reaction was stopped by adding 1.5 mL of DNS reagent and was then boiled for 10 min. Reaction mixture was allowed to cool to room temperature. Afterwards, all samples were centrifuged at 10,000 rpm for 5 min at 4 °C to free the reaction mixture of cell debris. Afterwards, OD was recorded at 540 nm. The xylanase activity was expressed as mg/mL by comparing with standard curve of xylose prepared while using 0–500 µg xylose.

### Cellulase activity

Cellulase activity was measured according to Ghose (1987). Seven hundred µl of 1 % carboxymethyl cellulose was incubated with 300 µl of CFCF. To stop the reaction, 1.5 ml of DNS reagent was added and boiled the reaction mixture for 10 min. After filtration, absorbance was measured at 540 nm. CMCase activity was expressed in mg/mL by comparing it with glucose standard curve prepared using 0–500 µg glucose.

### β-Xylosidase activity

The activity of β-xylosidase was estimated by Lachke’s method. Substrate selected for xylosidase activity was 4 mM solution of ρ-nitrophenyl-β-d-xylopyranoside (ρ-NPX). Nine hundred µL of *ρ*–NPX substrate along with 100µL of cell free culture filtrate was incubated at 50 °C for 30 min. Afterwards, 1 mL of 2 M sodium carbonate solution was added and absorbance was recorded at 410 nm. Standard curve of *ρ*–nitrophenol was used for comparison.

### Determination of total protein content

Bradford’s assay was used for total protein estimation. 1 mL of enzyme extract was taken in a glass tube and 3 mL of Bradford’s reagent was added to it. This mixture was incubated at room temperature for 5–10 min. Afterwards, OD was measured at 595 nm and compared against Bovine serum albumin (BSA) standard curve. Specific activity was calculated with the help of formula.

$$ {\text{Specific activity }}\left( {{\text{IU}}/{\text{mg}}} \right) = \frac{{{\text{Enzyme activity }}\left( {\text{IU}} \right)}}{{{\text{Protein content }}\left( {\text{mg}} \right)}} $$

### Phenotypic characterization of competent xylanolytic microbes

#### Molecular characterization

Potential xylanase producing microbial strains were identified on the basis of morphological, cultural, biochemical properties and ribotyping of conserved sequences. Molecular characterization of the best xylanase producing microbes was carried out to identify potential bacterial and fungal strains. Bacterial genomic DNA was extracted using phenol–chloroform extraction protocol (Sambrook and Russell 2001) whereas for fungal genomic DNA extraction, CTAB method was used. Afterwards, PCR amplification for the conserved sequences of DNA was performed and the amplified gene product was sequenced.

Molecular characterization of bacterial strains was carried out by amplifying 16S ribosomal RNA gene. Universal 16S rDNA primers were selected for gene amplification. 27F (5′-AGAGTTTGATCMTGGCTCAG-3′) and 1492R (5′-TACGGYTACCTTGTTACGACTT-3′) (Frank et al. 2008). Fungal rDNA ITS region was amplified while using ITS1 (5′-TCCGTAGGTGAACCTGCGG-3′) and ITS4 (5′-TCCTCCGCTTATTGATATGC-3′) (Fujita et al. 2001).

## Results

### Isolation and screening of xylanolytic strains

From all collected samples, a total of 184 microbial isolates were selected after primary screening on WBA medium. Out of these 184 isolates, 133 bacterial and 51 fungal strains were selected for secondary screening. Sixty-one bacterial and 27 fungal strains showed positive results to qualitative test of xylanase as indicated by the formation of clear zones in xylan agar medium plates.

### Xylanase production in SmF

The selected strains with clear zones in xylan agar plates were further subjected to enzyme diffusion technique for qualitative analysis of extracellular 1,4-β-endoxylanase production under submerged fermentation while using XBM. Cell free culture filtrate on incubation produced clear zones visible against opaque xylan intact agar in the medium. Zones of clearance were recorded before and after Congo red staining. Results were more or less the same as recorded before staining (Tables 2, 3).

### Quantification of xylanase production in cell free culture filtrate

**Fig. 1:**
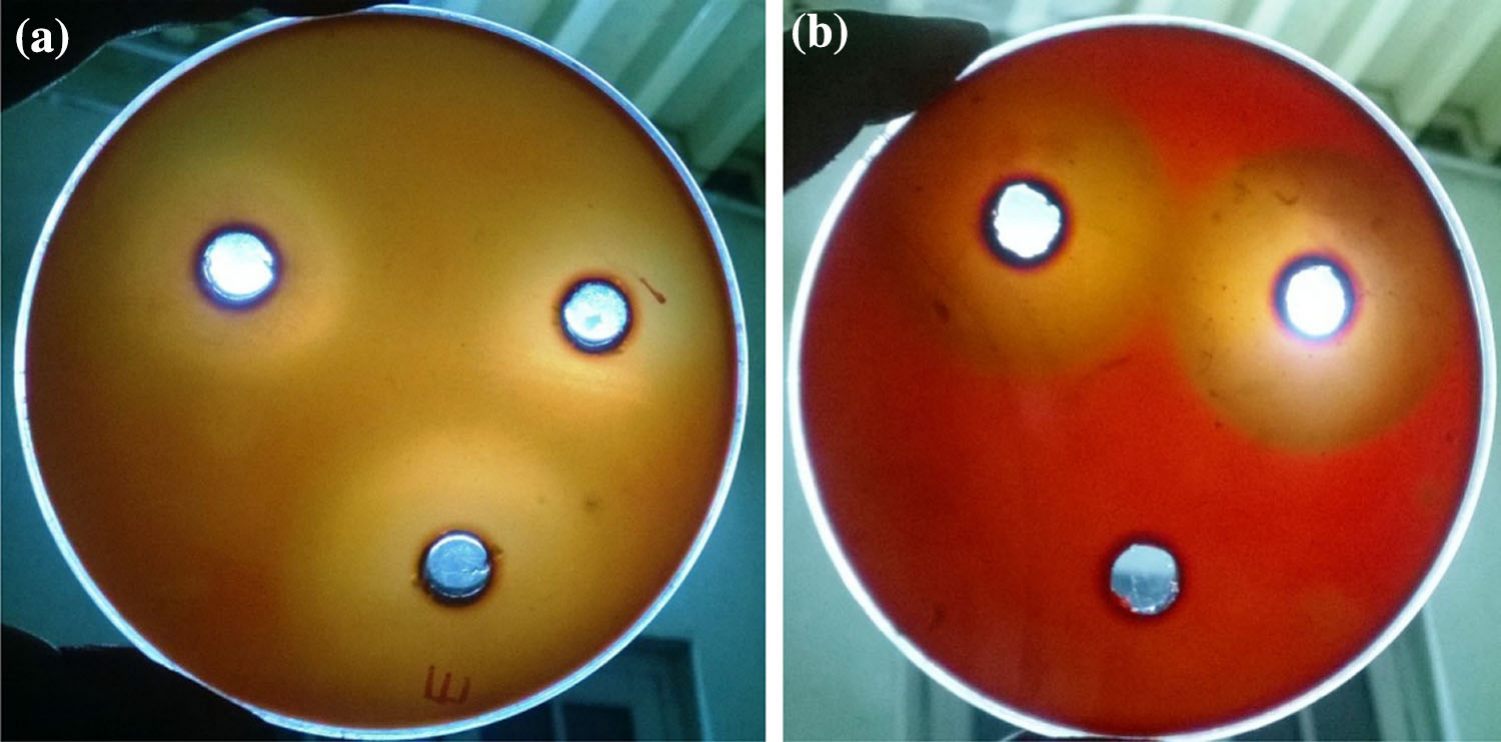
Screening of xylanolytic microbes **a** before and **b** after Congo red staining

**Fig. 2:**
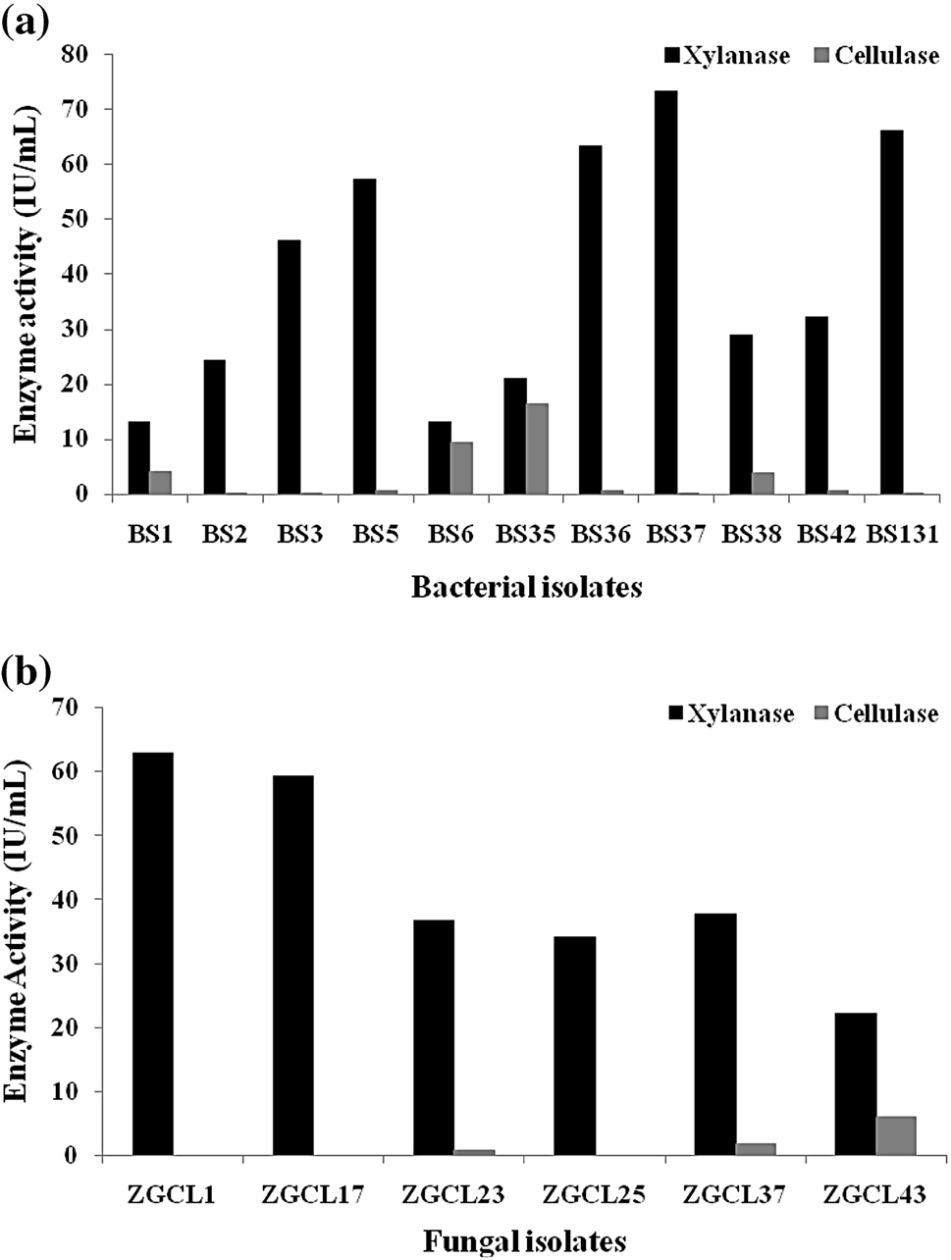
Xylanolytic and cellulolytic indexes of **a** bacterial and **b** fungal isolates

Cell free culture filtrate was assayed against DNS and color change in the mixture was observed at 553 nm. The eleven bacterial and six fungal isolates that have significant xylanase productions were presented in Figs. 1 and 2. It showed that bacterial isolates BS131, 37 and 3 were the best three for xylanolytic index, i.e., 388.82, 385.26 and 354.17 IU/ml, respectively. Whereas, among the fungal isolates, ZGCL17, ZGCL1 and ZGCL 25 presented relatively high xylanase indexes, i.e., 493.33, 419.33 and 391.82 IU/ml. Isolation sources of the best xylanolytic microbial strains are listed in table. Specific activity observed and compared with the xylose standard curve (Tables 1, 2).

**Table 1:** Qualitative and quantitative analysis of the extracellular enzyme produced by bacterial isolates

Sr. no.	Bacterial strain	Diameter of zone of clearance (mm) at different pH						Dry weight (mg)	Enzyme activity (IU/mL)				Total protein (mg/mL)	Specific activity (IU/mg)
		Simple diffusion			Congo red assay				Endoxylanase	CM case	FPase	β-Xylosidase		Endo-xylanase
		4.5	5.5	6.5	4.5	5.5	6.5							
1	BS131	32	30	30	32	30	31	1.7	66.1	0.01	0.43	8.12	0.17	388.82
2	BS37	31	29	31	31	29	31	2.1	73.2	0.06	0.04	5.12	0.19	385.26
3	BS3	25	24	22	25	26	22	1.1	46.1	0.02	0.34	5.18	0.13	354.17
4	BS2	19	23	20	21	23	22	1.2	24.4	0.09	0.63	9.12	0.07	348.57
5	BS5	19	20	24	19	20	25	3.2	57.3	0.49	0.31	8.17	0.19	301.58
6	BS42	21	19	20	21	19	22	4.5	32.2	0.38	0.09	8.34	0.11	292.73
7	BS1	18	17	21	18	19	23	1.3	13.3	3.92	11.2	15.8	0.05	266.00
8	BS38	19	18	22	21	18	22	2.4	29.1	3.63	3.51	6.26	0.11	264.55
9	BS36	17	19	16	17	19	17	2.1	63.3	0.44	0.13	7.35	0.27	234.44
10	BS6	19	16	21	19	16	21	1.3	13.2	9.21	7.21	3.35	0.06	220.00
11	BS35	15	21	22	15	20	22	2.7	21.2	16.2	19.3	2.63	0.11	192.73
12	BS73	11	11	10	11	11	10	1.3	8.45	7.46	11.3	10.7	0.07	120.71
13	BS92	11	12	10	11	11	09	2.2	5.13	9.17	13.2	5.78	0.06	85.50
14	BS123	09	08	09	09	08	09	1.1	7.91	8.43	9.18	9.33	0.13	60.851
15	BS27	07	09	09	07	09	08	1.5	9.52	6.74	9.13	8.77	0.17	56.00
16	BS65	07	07	08	07	07	08	2.1	11.8	9.91	13.2	5.83	0.23	51.300
17	BS78	05	05	06	05	05	07	1.5	3.52	14.1	13.8	15.4	0.09	39.11
18	BS99	06	05	06	06	05	07	2.1	4.94	8.32	9.87	11.9	0.16	30.88

**Table 2:** Qualitative and quantitative analysis of the extracellular enzyme produced by fungal isolates

Sr. no.	Fungal strain	Diameter of zone of clearance (mm) at different pH						Dry weight (mg)	Enzyme activity (IU/mL)				Total protein (mg/mL)	Specific activity (IU/mg)
		Simple diffusion			Congo red assay				Endoxylanase	CM case	FPase	β-Xylosidase		Endo-xylanase
		4.5	5.5	6.5	4.5	5.5	6.5							
1	ZGCL17	35	37	41	35	37	41	3.8	59.2	0.06	0.03	15.2	0.12	493.33
2	ZGCL1	38	35	37	39	36	37	5.4	62.9	0.01	0.05	9.13	0.15	419.33
3	ZGCL25	36	38	36	36	38	37	4.7	34.1	0.03	0.04	27.5	0.11	391.82
4	ZGCL37	29	26	31	30	26	31	3.9	37.7	1.81	3.72	18.72	0.12	284.16
5	ZGCL23	24	26	25	23	26	26	7.6	36.7	0.89	1.61	7.91	0.21	174.76
6	ZGCL43	18	18	15	18	19	15	4.2	22.2	5.94	7.54	3.21	0.13	170.77
7	ZGCL12	16	19	17	16	18	17	8.2	27.3	7.63	8.71	9.21	0.17	160.58
8	ZGCL15	11	10	13	11	13	13	9.7	29.8	2.32	4.83	9.12	0.19	156.84
9	ZGCL49	10	10	11	10	12	11	4.1	20.1	11.2	9.91	7.45	0.14	143.57
10	ZGCL33	13	11	12	13	11	12	9.3	18.9	13.2	17.2	11.2	0.19	99.47
11	ZGCL22	09	10	08	10	13	10	4.7	13.2	21.2	12.3	23.3	0.15	88.00
12	ZGCL11	11	08	12	11	08	11	11.3	19.9	11.9	13.4	5.18	0.32	62.19
13	ZGCL3	07	09	08	07	11	10	9.2	18.9	19.7	11.8	19.3	0.29	65.17
14	ZGCL41	07	08	10	07	10	10	12.1	11.9	17.6	9.31	19.7	0.32	37.19
15	ZGCL4	05	07	07	06	07	07	16.1	33.9	16.4	11.3	2.87	1.1	30.82

Various assays were performed to estimate the production of different hydrolytic enzymes. The 17 potential isolates were subjected to test for their xylanase activity. Activities of xylanase, cellulase, β-xylosidase were estimated using appropriate protocols. The total protein content was determined by Bradford’s method (Tables 1, 2) (Bradford 1976; Lachke 1988; Miller 1959; Zhang et al. 2011).

### Phenotypic characterization of competent xylanolytic microbes

Results were recorded for bacterial & fungal strains while using Bergey’s manual and listed in table (Table 3). All microbial strains were identified using rRNA gene sequencing and their resemblance with the closest type strains are presented in Tables 4, 5. The best xylanolytic bacterial strain BS131 was identified as *Bacillus pumilus* and the best fungal producer ZGCL17 was identified as *Aspergillus flavus*.

**Table 3:** Morphological Identification of potential xylanase producing microbes

Fungal strain	Isolation source	Identified as
ZGCL1	Soil	*Aspergillus* sp.
ZGCL17	Soil	*Aspergillus* sp.
ZGCL23	Saw dust	*Fusarium* sp.
ZGCL25	Soil	*Penicillium* sp.
ZGCL37	Decaying wood	*Trichoderma sp.*
ZGCL43	Soil	*Aspergillus* sp.
BS1	Ruminal fluid (Goat)	*Bacillus* sp.
BS2	Decaying cow dung	*Bacillus* sp.
BS3	Leaf compost	*Bacillus* sp.
BS5	River bank soil	*Providencia* sp.
BS6	Paper mill effluent	*Bacillus* sp.
BS35	Raw milk	*Staphylococcus* sp.
BS36	Moist soil	*Bacillus* sp.
BS37	Moist soil	*Bacillus* sp.
BS38	Decaying agro-waste	*Bacillus* sp.
BS42	Leaf compost	*Bacillus* sp.
BS131	Moist soil	*Bacillus* sp.

**Table 4:** Identification of potential xylanase producing bacteria based on 16S ribosomal RNA gene sequence

Sr. no.	Bacterial strain	Isolation source	Identified as	Similarity (%)	NCBI Accession no.
1	BS1	Ruminal fluid (Goat)	*Bacillus cereus*	100	KT356279
2	BS2	Decaying cow dung	*Bacillus altitudinis*	100	KT381614
3	BS3	Leaf compost	*Bacillus cereus*	100	KT356281
4	BS5	River bank soil	*Providencia rettgeri*	99	KT381615
5	BS6	Paper mill effluent	*Bacillus amyloliquefaciens*	99	KT381616
6	BS35	Raw milk	*Staphylococcus warneri*	99	KX189101
7	BS36	Moist soil	*Bacillus pumilus*	100	KT583750
8	BS37	Moist soil	*Bacillus safensis*	100	KT354645
9	BS38	Decaying agro-waste	*Bacillus pumilus*	99	KT962916
10	BS42	Leaf compost	*Bacillus subtilis*	99	KT721566
11	BS131	Moist soil	*Bacillus pumilus*	100	KT962917

**Table 5:** Identification of potential xylanase producing fungi based on ITS gene sequence

Sr. no.	Fungal strain	Isolation source	Identified as	Similarity (%)	NCBI Accession no.
1	ZGCL1	Soil	*Aspergillus niger*	99	KT970477
2	ZGCL17	Soil	*Aspergillus flavus*	99	KT970478
3	ZGCL23	Saw dust	*Fusarium oxysporum*	99	KT970482
4	ZGCL25	Soil	*Penicillium digitatum*	99	KT970480
5	ZGCL37	Decaying wood	*Trichoderma harzianum*	99	KT970481
6	ZGCL43	Soil	*Aspergillus oryzae*	99	KT970479

## Discussion

Out of the eleven bacterial and six fungal isolates that have been characterized for their cellulase free xylanase activity, *Bacillus pumilus* (BS131) and *Bacillus safensis* (BS37) are potential bacterial isolates due to their significant xylanase and minimum cellulase production. *Aspergillus flavus* (ZGCL17) and *Aspergillus niger* (ZGCL1) are the best cellulase free xylanase producers among the fungal isolates. These isolates have potential to be used in industrial processes especially suitable for paper and pulp industries.

Hence, isolation, screening and selection have facilitated the discovery of several cellulase free xylanase producers from a wide variety of environmental samples. The results indicate possible employment of such enzymes in a number of industrial processes with a decrease in current cost of bioconversion of lignocellulosic mass.
